# Central Nervous System Lymphoma in a Patient with Chronic Lymphocytic Leukemia: A Case Report and Literature Review

**DOI:** 10.7759/cureus.3660

**Published:** 2018-11-30

**Authors:** Abdulrahman Albakr, Wajda Alhothali, Peyman Samghabadi, Lauren Maeda, Seema Nagpal, Abdulrazag Ajlan

**Affiliations:** 1 Neurosurgery, University of Calgary/ Foothills Medical Center, Calgary, CAN; 2 Neurosurgery, King Saud University, Riyadh, SAU; 3 Pathology, Stanford University School of Medicine, Stanford, USA; 4 Internal Medicine, Stanford University School of Medicine, Stanford, USA; 5 Neurology, Stanford University School of Medicine, Stanford, USA; 6 Neurosurgery, Stanford University School of Medicine, Stanford, USA

**Keywords:** central nervous system, chronic lymphocytic leukemia, neurologic involvement, primary central nervous system lymphoma

## Abstract

Chronic lymphocytic leukemia (CLL) is the most common type of leukemia that affects older adults in the Western world. Symptomatic nervous system invasion in undiagnosed CLL is rare, poorly understood, challenging to treat, and associated with decreased survival. The average survival of CLL patients with central nervous system (CNS) involvement is 3.79 years as compared to six years in CLL patients without CNS involvement. Autopsy studies demonstrated a high incidence of undiagnosed CLL with CNS involvement, suggesting that CNS involvement is either underdiagnosed or subclinical. Although the most common site of CNS involvement is the leptomeninges, our case demonstrates an extremely rare form of CNS diffuse large B-cell parenchymal involvement in a patient with a concurrent diagnosis of systemic CLL.

## Introduction

The incidence of central nervous system (CNS) involvement in chronic lymphocytic leukemia (CLL) ranges from 0.8% to 2% in antemortem studies and up to 7% to 71% of cases diagnosed at autopsy [1-2]. Skin, lung, and kidney are other organs that can be affected by CLL [3]. CNS involvement with CLL has a non-specific spectrum of symptoms and signs and no apparent correlation with patient gender, age, presentation, duration of the disease, and Rai stage at the time of diagnosis [3]. The radiological features of CNS involvement have been described as a diffuse coating of the leptomeninges with the infiltration of the parenchyma [3].

Richter’s syndrome is the progression of CLL to high-grade non-Hodgkin lymphoma, with an incidence of 5% [4]. Studies have shown that Richter’s syndrome is considered a risk factor predisposing to CNS involvement [5]. Here, we report a case of CNS lymphocytic intra-parenchymal involvement in a patient with previously undiagnosed CLL.

## Case presentation

**Figure 1:**
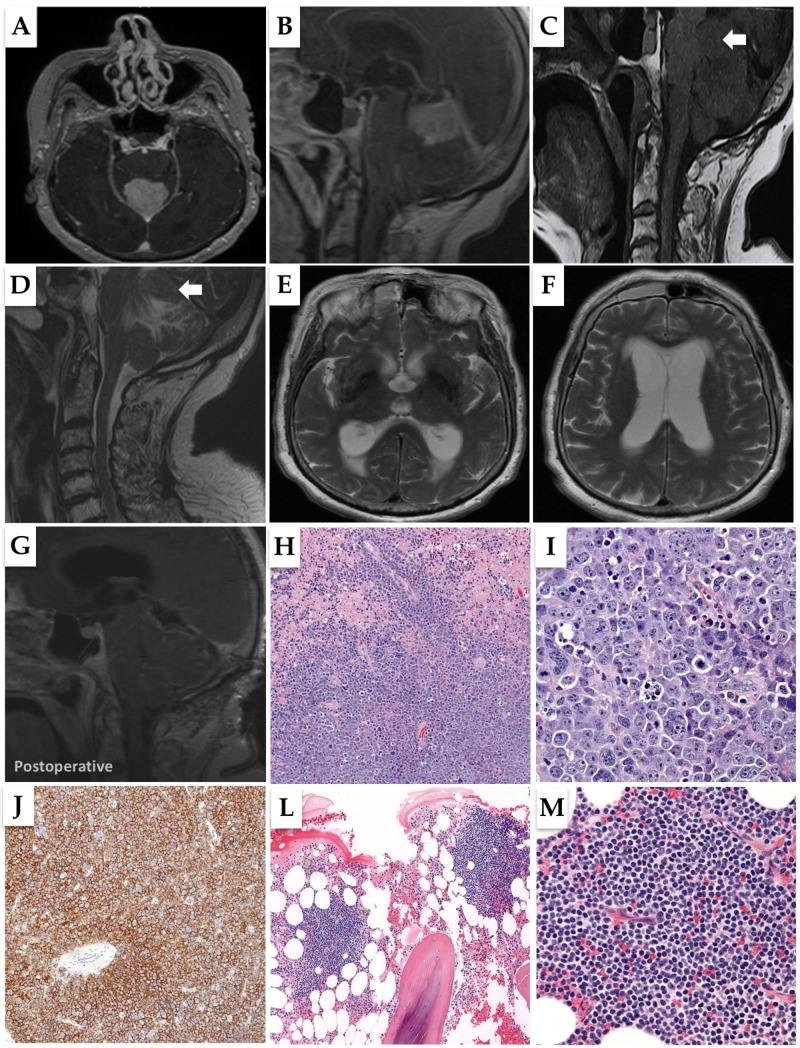
Preoperative brain MRI and histopathological studies of the resection specimen (1A, 1B) Preoperative axial and sagittal sections, post-contrast T1 magnetic resonance imaging (MRI) of the brain, showing a midline, supra-vermilion homogeneously enhancing lesion causing a significant mass effect and obstruction on the aqueduct. (1C, 1D) Preoperative sagittal sections, T1 and T2 MRI of the brain showing an isointense lesion on both sequences (arrow). (1E, 1F) Preoperative axial sections, T2 magnetic resonance imaging (MRI) of the brain showing dilated third and lateral ventricles. (1G) Postoperative sagittal T1 magnetic resonance imaging (MRI) of the brain. (1H) Low power view of the resection specimen revealing an infiltrating, angiocentric neoplasm associated with abundant necrosis (Hematoxylin and Eosin, 100X). (1I) High power view demonstrating mitotically active, malignant lymphoid cells, with open chromatin, nuclear membrane irregularities, and prominent-often multiple-nucleoli (Hematoxylin and Eosin, 400X). (1J) Immunohistochemistry for CD20 was diffusely positive (100X). (1L) Low power view of the bone marrow core biopsy revealing involvement by well-circumscribed, non-paratrabecular lymphoid aggregates (Hematoxylin and Eosin, 100X). (1M) High power view demonstrating a monomorphic population of small to medium-sized lymphoid cells with mild cytologic atypia (Hematoxylin and Eosin, 400X)

An 84-year-old woman, known to have atrial fibrillation and hypertension, presented with impaired memory and altered mental status. On physical examination, no lymphadenopathy or organomegaly was detected. A neurological examination revealed mild dysmetria in the left upper extremity. Her white cell count was 25,100 × 10^12^/l, with 61% lymphocytes. Magnetic resonance imaging (MRI) of the brain revealed a homogeneously enhancing cerebellar mass causing mass effect on the tectum and obstruction at the level of the aqueduct associated with the hydrocephalus (Figures 1A-1F).

The patient underwent a bilateral posterior fossa craniotomy and tumor resection, followed by the insertion of an external ventricular drain. Intraoperatively, the mass was thought to be intra-parenchymal. After surgery, she recovered well, with no new neurological deficits. Histopathology revealed an infiltrating and highly mitotic neoplasm composed of malignant lymphoid cells (Figures 1H-1J). In situ hybridization (ISH) for Epstein–Barr virus (EBV) was negative. Fluorescence in situ hybridization (FISH) demonstrated no gene rearrangements in B-cell lymphoma 2 (BCL2), (BCL6), and MYC. Polymerase chain reaction (PCR) amplification and capillary gel electrophoresis per the BIOMED-2 protocol were performed on paraffin-embedded tissue, revealing a peak in the immunoglobulin heavy chain (IGH) consistent with a clonal process. The findings were diagnostic of an EBV negative, diffuse large B cell lymphoma (DLBCL).

A bone marrow core biopsy was performed due to low white blood cell (WBC) count, which revealed the involvement of a low-grade lymphoid process (Figures 1L-1M). Flow cytometric immunophenotyping revealed a Lambda-monotypic, CD5 negative B cell population expressing CD19, CD23, CD22 (dim), and partial CD20 while lacking CD10 and FMC7. Aside from the lack of CD5 expression, the morphologic and immunophenotypic findings were consistent with chronic lymphocytic leukemia/small cell lymphoma. A diagnosis of small B cell lymphoma was rendered, with an offered differential diagnosis including CLL, marginal zone lymphoma, mantle cell lymphoma (CD5 negative), follicular lymphoma (CD10 negative), and lymphoplasmacytic lymphoma. Although the lack of CD5 expression makes mantle cell lymphoma a more reasonable differential, multiple studies have reported cases of CD5 negative CLL [6-7], with an incidence ranging from 7% to 20% among all CLL cases [7]. Hence no further investigations to rule out mantle cell lymphoma were required.

As mentioned above, molecular studies performed on the paraffin-embedded tissue from the brain biopsy revealed an immunoglobulin heavy chain (IGH) rearrangement consistent with a clonal process. We endeavored to perform a similar analysis on a sample from the patient's bone marrow biopsy, as the presence of an identical gene rearrangement would have provided support for a relationship between the two neoplasms. However, per standard protocols, the bone marrow core biopsy was acid decalcified for next day processing, compromising the integrity of the DNA necessary for the PCR. Therefore, performing an IGH rearrangement studies on the bone marrow biopsy was not a viable option, and a definitive genetic link between the two neoplastic processes could not be established. 

The positron emission tomography/computed tomography (PET/CT) scan showed no other areas of hypermetabolic involvement. Given her advanced age, she might not have tolerated the toxic effect of methotrexate. Therefore, she received one cycle of temozolomide and whole-brain radiation therapy (WBRT). A follow-up CT scan at six months showed no residual tumor. Approximately 10 months following her initial diagnosis, she had a recurrence in the posterior fossa, which was confirmed by MRI. She passed away within one month of recurrence.

## Discussion

Symptomatic nervous system invasion in undiagnosed CLL is rare, poorly understood, and challenging to treat. Symptoms of CLL include weakness, weight loss, fever, night sweat, bleeding, infection, and pressure symptoms from enlarged lymph nodes [8]. The Rai staging system is one of several clinical staging systems that assess the clinical outcome for CLL patients and assigns a score based on the presence or absence of lymphocytosis, lymph node enlargement, splenomegaly, hepatomegaly, anemia, and thrombocytopenia [8]. Our patient was Rai stage 0 at the time of diagnosis.

CNS involvement in CLL can occur at any time during the disease course. Onset ranges from the time of the initial onset to 14 years after the diagnosis [5]. The average latency from the onset of CNS symptoms to mortality was reported in 36 patients as 12 months [3]. The average survival for patients with CNS involvement has been reported as 3.79 years from the time of CLL diagnosis to death, compared to CLL patients without CNS involvement, who have an average survival of six years [9].

The incidence of CNS involvement in CLL ranges from 0.8% to 2% [1]. Autopsy studies have demonstrated that CNS involvement ranges from 7% to 71% [2-3]. These numbers suggest that CNS involvement is frequently not diagnosed and remains subclinical. While CNS involvement in CLL is considered rare, the involvement of brain parenchyma is extremely rare [10]. Moreover, with the most common site of CNS involvement being the leptomeninges, a recent study that reviewed the reported cases of CLL with CNS involvement indicated that only nine out of 80 patients had a parenchymal infiltration, and only two patients among those who had parenchymal infiltration had CNS involvement upon presentation [3]. CNS involvement with lymphoma can present with non-specific symptoms. Commonly reported symptoms include visual changes 22%, encephalopathy 29%, and weakness with paresthesias 22% [9]. In a literature review of CLL with CNS involvement, only 32 out 80 of patients had proven imaging findings [3]. Therefore, our case demonstrates an extremely rare form of CNS intra-parenchymal involvement in a previously undiagnosed CLL patient and, to our knowledge, is the third reported case of undiagnosed CLL presenting with CNS intra-parenchymal infiltration.

The incidence of CNS involvement in patients with Richter’s syndrome is unknown. One study reported five of 39 patients with Richter’s syndrome having CNS involvement at the time of the initial presentation [4]. The incidence of CNS involvement in non-transformed CLL is rare [11]. The mechanisms involved in this transformation are not well understood. The expression of CD38, deletion of 13q14, and predominant nodal disease are associated with an increased risk of CLL progression [11]. Richter’s syndrome can be associated with a marked elevation of lactate dehydrogenase (LDH) level, rapidly enlarged lymph nodes, and fever in the absence of infection [4]. Our patient did not show any clinical symptoms of Richter’s syndrome. The other differential diagnoses include a CLL with coincidental, de novo primary central nervous system lymphoma (PCNSL).

In some reports, it has been suggested that CNS involvement occurs at an advanced disease and late Rai stages [12]. Nonetheless, most of the studies suggest that there is no clear association between the Rai stage of the disease and the CNS involvement [1,11]. One study reported that 18 out of 80 patients with CLL and CNS involvement were associated with Rai stage 0 [3]. Another review of 25 cases of CLL with CNS involvement showed that nine of the cases were associated with early stages of CLL at the time of CNS involvement [5]. The current literature suggests that CNS involvement in CLL is not related to Rai stage, progression, or duration of the disease [10].

Due to the rarity of this condition, there is no standard of care for the treatment of CNS involvement in CLL. The most frequently used therapy described in the literature consists of intrathecal chemotherapy, radiation therapy, and/or systemic chemotherapy. Corticosteroids and intrathecal chemotherapy such as methotrexate and cytarabine are usually the mainstay treatment of leptomeningeal diseases in CLL [9]. Radiation therapy appears to have a good response in cases with cerebral infiltration [3]. In one review, six patients who received radiation therapy for the intraparenchymal disease were described; five of them showed the resolution of intraparenchymal infiltrations and resolution of CNS symptoms, with a survival rate that ranged from three months to three years [3]. Despite treatment, the prognosis for patients with clinically significant CNS involvement in CLL remains poor [13-14]. Our patient was treated with surgery, followed by one cycle of temozolomide and WBRT.

## Conclusions

CLL with CNS involvement at initial presentation is an uncommon manifestation. CNS involvement should be considered when patients with CLL present with neurologic disturbances. Other differential diagnoses include a CLL with unrelated PCNSL or CLL secondary involvement in the context of Richter’s syndrome. We report the case of a patient with previously undiagnosed CLL presenting with an intra-parenchymal posterior fossa diffuse large B-cell parenchymal tumor. A definitive genetic link between CLL and PCNSL in similar reports should be confirmed in future cases.
